# ERG oncoprotein expression in prostatic acinar adenocarcinoma; clinicopathologic significance

**DOI:** 10.1186/s13104-019-4090-x

**Published:** 2019-01-18

**Authors:** Atif Ali Hashmi, Erum Yousuf Khan, Muhammad Irfan, Rabia Ali, Huda Asif, Maheen Naeem, Laila Nisar, Naveen Faridi, Amir Khan, Muhammad Muzzammil Edhi

**Affiliations:** 1 Liaquat National Hospital and Medical College, Karachi, Pakistan; 2 CMH Institute of Medical Sciences, Multan, Pakistan; 3 Kandahar University, Kandahar, 3802 Afghanistan; 4 Brown University, Providence, RI USA

**Keywords:** ERG oncoprotein, Prostatic adenocarcinoma, Gleason score, WHO grade group, Immunohistochemistry

## Abstract

**Objectives:**

T/E fusion results in constitutive expression of ERG oncoprotein resulting in enhanced proliferation and invasive potential of prostatic cancer cells. In the present study we aimed to evaluate the ERG overexpression in 78 cases prostate acinar adenocarcinoma and its association with other prognostic parameters.

**Results:**

ERG protein expression was noted in 39.7% (31 cases), out of which 3 cases (3.8%) showed low ERG expression, 10 cases (12.8%) showed intermediate expression and 18 cases (23.1%) revealed high ERG expression. Significant association of ERG expression was noted with gleason score (p = 0.009), WHO grade group (p = 0.008) and perineural invasion (p = 0.043). We found a significant proportion of our patients of prostatic acinar adenocarcinoma to over-express ERG protein which can help in devising therapeutic protocols. Significant association of ERG protein expression with gleason score and perineural invasion signifies its prognostic significance in prostatic carcinoma. Moreover, we also suggest that molecular studies should be performed in patients with prostatic carcinoma to look for T/E fusion gene and its correlation with ERG protein expression.

## Introduction

Prostate cancer is one of the most common malignancies in males. Recently, it has been suggested that TMPRSS2/ERG (T/E) fusion gene is present in approximately 50% of prostatic adenocarcinoma [1]. T/E fusion results in constitutive expression of ERG oncoprotein resulting in enhanced proliferation and invasive potential of prostatic cancer cells [2–6]. Moreover, T/E fusion gene product can be an important therapeutic target in prostatic cancer. Immunohistochemical (IHC) overexpression of ERG oncoprotein may serve as a surrogate biomarker of T/E fusion gene. Therefore in the present study we aimed to evaluate the ERG overexpression in prostate acinar adenocarcinoma and its association with other prognostic parameters.

## Main text

### Patients and methods

The study was conducted at Liaquat National hospital Karachi, incorporating 78 biopsy proven cases of prostatic acinar adenocarcinoma. Ethics committee of the hospital approved the study, while informed written consent from the patients was taken from the patients before surgery. After surgery, specimens of transurethral resections and radical prostatectomies were sent to histopathology department. Clinical characteristics of the patients were recorded from patient files and histopathologic characteristics were re-evaluated by reviewing histopathologic slides. For ERG immunohistochemistry, representative tissue blocks were selected.

### ERG immunohistochemistry

ERG immunohistochemistry was performed using ERG (EP111) antibody purchased from Cell Marque and DAKO EnVision kit according to manufacturers protocol. IHC staining was performed manually in batches of 10 slides. Positive (k/c prostatic carcinoma) and negative (normal prostatic tissue) controls were run along with each batch. For IHC staining, 3–4 µm sections were taken on DAKO IHC coated slides. After fixation of slides in oven at 70–80 °C for 20 min, sections were de-waxed in xylene and then through decreasing concentrations of alcohol. Slides were then placed in antigen retrieval solution in water bath at 99–100 °C for 40 min. After being kept at room temperature, slides were washed with wash buffer solution 2–3 times, followed by blocking peroxidase for 10 min in humidity chamber. Subsequently, slides were washed 2–3 times with wash buffer. Primary antibody (ERG) was applied with dilution of 1:200 for 20 min, then washed 2–3 times. After application of secondary antibody, slides were incubated for 20 min and then again washed with wash buffer, followed by application of DAB chromogen solution.

**Fig. 1:**
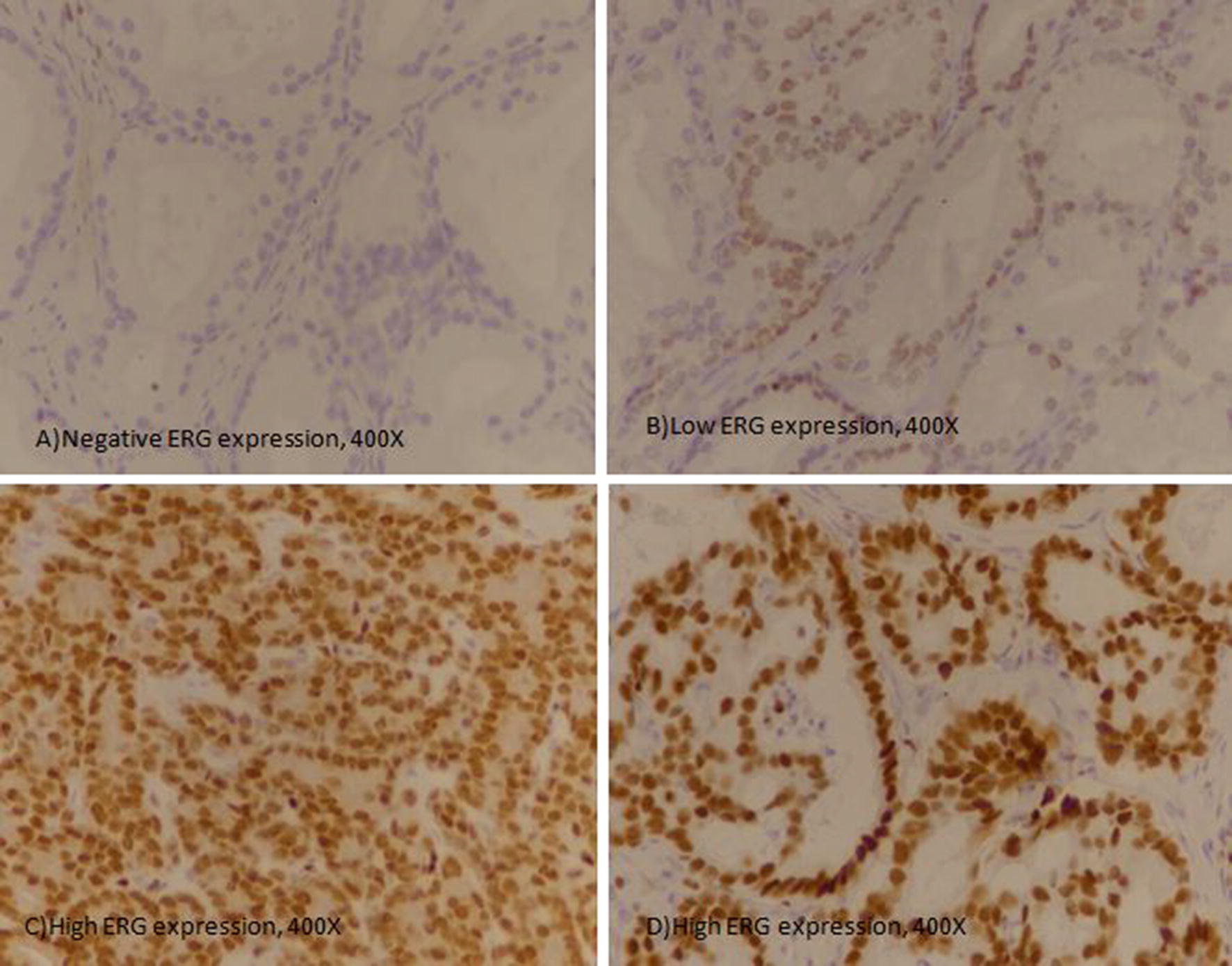
ERG expression in prostatic adenocarcinoma

Nuclear staining for ERG was quantitatively and qualitatively evaluated. Intensity of staining was scored into no staining (0), weak (1 +), intermediate (2 +), strong (3 +) while percentage of positively stained cells were scored as continuous variable (Fig. 1). Intensity and percentage scores were multiplied to generate an H-score ranging from 0 to 300. Cases with < 10 score was categorized as no ERG expression, 11–100 score was taken as low ERG expression, 101–200 score was categorized as intermediate ERG expression while > 200 score was taken as high ERG expression.

### Patient characteristics

Mean patients age was 69.02 ± 8.82 years. Low gleason score i.e. 6 and 7 were noted in 25.6% (20 cases) and 29.5% (23 cases) respectively, whereas high gleason score i.e. 8 and 9 were noted in 5.1% (4 cases) and 39.7% (31 cases) respectively. Similarly, high WHO grade group i.e. 4 and 5 were seen in 5.1% (4 cases) and 39.7% (31 cases) respectively. > 50% tissue involvement by tumor (tumor quantification) was noted in 55.1% (43 cases). Perineural invasion was seen in 32.1% (25 cases). Lymphovascular invasion, extraprostatic extention and seminal vesicle invasion was noted in 1.3%, 6.4% and 5.1% cases respectively (Table 1).

**Table 1:** Clinicopathologic characteristics of studied population (n = 78)

	n (%)
Age (years)	
Mean	69.02 ± 8.82
Groups (years)	
< 40	0 (0)
40–70	49 (62.8)
> 70	29 (37.2)
Tumor quantification	
Mean	50.91 ± 30.09
Groups (%)	
< 10	11 (14.1)
10–50	24 (30.8)
> 50	43 (55.1)
Total gleason score	
6	20 (25.6)
7	23 (29.5)
8	4 (5.1)
9	31 (39.7)
Grade	
Grade I	20 (25.6)
Grade II	4 (5.1))
Grade III	19 (24.4)
Grade IV	4 (5.1)
Grade V	31 (39.7)
Tumor quantification	
< 10	11 (14.1)
10–50	24 (30.8)
> 50	43 (55.1)
Perineural invasion	
Present	25 (32.1)
Absent	53 (67.9)
Lymphovascular invasion	
Present	1 (1.3)
Absent	77 (98.7)
Extraprostatic extension	
Present	5 (6.4)
Absent	73 (93.6)
Seminal vesicle invasion	
Present	4 (5.1)
Absent	74 (94.9)
ERG score	
Mean	81.02 ± 111.64
Groups	
No expression	47 (60.3)
Low expression	3 (3.8)
Intermediate expression	10 (12.8)
High expression	18 (23.1)

### ERG oncoprotein expression

ERG protein expression was noted in 39.7% (31 cases), out of which 3 cases (3.8%) showed low ERG expression, 10 cases (12.8%) showed intermediate expression and 18 cases (23.1%) revealed high ERG expression. Significant association of ERG expression was noted with gleason score (p = 0.009), WHO grade group (p = 0.008) and perineural invasion (p = 0.043) (Table 2).

**Table 2:** Association of ERG expression with clinicopathologic parameters in prostatic acinar adenocarcinoma

	n (%)				*p* value
	No expression (n = 47)	Low expression (n = 3)	Intermediate expression (n = 10)	High expression (n = 18)	
Age group (years)					
40–70	30 (63.8)	0 (0)	5 (50)	14 (77.8)	0.059
> 70	17 (36.2)	3 (100)	5 (50)	4 (22.2)	
Total gleason score					
6	18 (38.3)	0 (0)	1 (10)	1 (5.6)	0.009
7	14 (29.8)	1 (33.3)	4 (40)	4 (22.2)	
8	2 (4.3)	0 (0)	2 (20)	0 (0)	
9	13 (27.7)	2 (66.7)	3 (30)	13 (72.2)	
Grade					
Grade I	18 (38.3)	0 (0)	1 (10)	1 (5.6)	0.008
Grade II	2 (4.3)	0 (0)	0 (0)	2 (11.1)	
Grade III	12 (25.5)	1 (33.3)	4 (40)	2 (11.1)	
Grade IV	2 (4.3)	0 (0)	2 (20)	0 (0)	
Grade V	13 (27.7)	2 (66.7)	3 (30)	13 (72.2)	
Tumor quantification					
< 10	10 (21.3)	0 (0)	0 (0)	1 (5.6)	0.398
10–50	14 (29.8)	0 (0)	3 (30)	7 (38.9)	
> 50	23 (48.9)	3 (100)	7 (70)	10 (55.6)	
Perineural invasion					
Present	10 (21.3)	1 (33.3)	6 (60)	8 (44.4)	0.043
Absent	37 (78.7)	2 (66.7)	4 (40)	10 (55.6)	
Lymphovascular invasion					
Present	1 (2.1)	0 (0)	0 (0)	0 (0)	1.000
Absent	46 (97.9)	3 (100)	10 (100)	18 (100)	
Extraprostatic extension					
Present	2 (4.3)	0 (0)	0 (0)	3 (16.7)	0.357
Absent	45 (95.7)	3 (100)	10 (100)	15 (83.3)	
Seminal vesicle invasion					
Present	2 (4.3)	0 (0)	0 (0)	2 (11.1)	0.534
Absent	45 (95.7)	3 (100)	10 (100)	16 (88.9)	

Fisher exact test applied

p-value ≤ 0.05, considered as significant

## Discussion

In the present study, we evaluated the ERG protein expression in 78 cases of prostatic acinar adenocarcinoma. A significant proportion of cases of prostatic acinar adenocarcinoma in our studied population were found to have ERG protein overexpression. It has been noted in previous literature that IHC overexpression of ERG protein strongly correlates with the presence of T/E fusion gene [7]. A few studies aimed to evaluate the prognostic significance of ERG protein expression and reported both positive and negative associations with other prognostic parameters [8, 9]. Furusato et al. found that ERG overexpression was associated with high gleason score [7]. Similarly, in the present study we found a significant association of ERG protein expression with gleason score/WHO grade group and perineural invasion which are the most important prognostic parameters in prostatic adenocarcicoma.

The Expression Status of ERG in prostate cancer has been extensively studies in prostate cancer specimens. Wang et al. found that T/E fusion was present in approximately half of the cases of prostatic carcinoma (n = 200). They found that T/E fusion gene isoforms differentially increase NF-κB-mediated transcription, thus promoting prostatic cancer proliferation and invasion [10]. Similar, the association between the ERG rearrangement and the expression status of ERG in prostate cancer using antibody-based detection has been demonstrated previously in various studies. Mcleod et al. utilizing PCa tissue microarrays from 207 patients, established a correlation between the presence of ERG protein expression by immunohistochemistry (IHC) and *ERG* rearrangement by using fluorescence in situ hybridization (FISH) with a sensitivity and specificity of 95.7% and 96.5% respectively [11]. Park et al. found that expression of ERG was present in endothelial cells of small vessels in both benign and cancerous prostate tissues and in lymphocytes of both benign and cancerous tissues. Furthermore, Park et al., reported no association between the ERG rearrangement status and clinical or pathologic parameters using two cohorts, the Weill Cornell Medical college cohort (n = 128) and University of Michigan cohort (n = 79) respectively [12].

## Limitations

Our study was limited due to small number of cases, non availability of molecular studies to evaluate T/E gene fusion status and lack of clinical followup. However, we found a significant proportion of our patients of prostatic acinar adenocarcinoma to overexpress ERG protein which can help in devising therapeutic protocols. Moreover, we also suggest that molecular studies should be performed in patients with prostatic carcinoma to look for T/E fusion gene and its correlation with ERG protein expression.

## Abbreviation

IHC: immunohistochemical
